# Salubrinal Enhances Cancer Cell Death during Glucose Deprivation through the Upregulation of xCT and Mitochondrial Oxidative Stress

**DOI:** 10.3390/biomedicines9091101

**Published:** 2021-08-28

**Authors:** Mei-Chun Chen, Li-Lin Hsu, Sheng-Fan Wang, Yi-Ling Pan, Jeng-Fan Lo, Tien-Shun Yeh, Ling-Ming Tseng, Hsin-Chen Lee

**Affiliations:** 1 Department and Institute of Pharmacology, College of Medicine, National Yang Ming Chiao Tung University, Taipei 112, Taiwan; mcchen23@vghtpe.gov.tw (M.-C.C.); lindahsu0807@yahoo.com.tw (L.-L.H.); sfwang5@vghtpe.gov.tw (S.-F.W.); drivten10@gmail.com (Y.-L.P.); jflo@nycu.edu.tw (J.-F.L.); 2 Division of Plastic and Reconstructive Surgery, Department of Surgery, Taipei Veterans General Hospital, Taipei 112, Taiwan; 3 Department of Surgery, College of Medicine, National Yang Ming Chiao Tung University, Taipei 112, Taiwan; 4 Department of Pharmacy, Taipei Veterans General Hospital, Taipei 112, Taiwan; 5 Department of Clinical Pharmacy, School of Pharmacy, Taipei Medical University, Taipei 110, Taiwan; 6 Department of Dentistry, College of Dentistry, National Yang Ming Chiao Tung University, Taipei 112, Taiwan; 7 Institute of Oral Biology, National Yang Ming Chiao Tung University, Taipei 112, Taiwan; 8 Cancer Progression Research Center, National Yang Ming Chiao Tung University, Taipei 112, Taiwan; 9 Institute of Anatomy and Cell Biology, College of Medicine, National Yang Ming Chiao Tung University, Taipei 112, Taiwan; tsyeh@nycu.edu.tw; 10 Comprehensive Breast Health Center, Department of Surgery, Taipei Veterans General Hospital, Taipei 112, Taiwan; 11 Department of Pharmacy, College of Pharmaceutical Sciences, National Yang Ming Chiao Tung University, Taipei 112, Taiwan

**Keywords:** integrated stress response, salubrinal, oxidative stress, xCT

## Abstract

Cancer cells have the metabolic flexibility to adapt to heterogeneous tumor microenvironments. The integrated stress response (ISR) regulates the cellular adaptation response during nutrient stress. However, the issue of how the ISR regulates metabolic flexibility is still poorly understood. In this study, we activated the ISR using salubrinal in cancer cells and found that salubrinal repressed cell growth, colony formation, and migration but did not induce cell death in a glucose-containing condition. Under a glucose-deprivation condition, salubrinal induced cell death and increased the levels of mitochondrial reactive oxygen species (ROS). We found that these effects of salubrinal and glucose deprivation were associated with the upregulation of xCT (SLC7A11), which functions as an antiporter of cystine and glutamate and maintains the level of glutathione to maintain redox homeostasis. The upregulation of xCT did not protect cells from oxidative stress-mediated cell death but promoted it during glucose deprivation. In addition, the supplementation of ROS scavenger N-acetylcysteine and the maintenance of intracellular levels of amino acids via sulfasalazine (xCT inhibitor) or dimethyl-α-ketoglutarate decreased the levels of mitochondrial ROS and protected cells from death. Our results suggested that salubrinal enhances cancer cell death during glucose deprivation through the upregulation of xCT and mitochondrial oxidative stress.

## 1. Introduction

Cancer cells are rapid-proliferation cells and need a large amount of nutrients to support their production of energy and macromolecules. During the rapid growth of tumors, the tumor microenvironment is usually under the conditions of hypoxia and low levels of nutrients due to the irregular new vascular network with poor perfusion [1,2]. To adapt to the heterogeneous tumor microenvironment, the characteristics of metabolic flexibility and plasticity are essential for cancer cells [3]. The question of how cancer cells adapt to nutrient deprivation and regulate their metabolic flexibility to maintain cell survival is still poorly understood.

The integrated stress response (ISR) is an adaptive signaling pathway in eukaryotic cells that helps cells to maintain cellular homeostasis. Several stressors, including endoplasmic reticulum (ER) stress, nutrient deprivation, viral infection, and reduced heme levels, can activate ISR [4,5,6,7]. ISR attenuates protein synthesis but promotes the translation of the activation of transcription factor 4 (ATF4), a main downstream regulator of ISR, through the phosphorylation of the alpha subunit of eukaryotic translation initiation factor 2 (eIF2α) [8]. ATF4 regulates many stress response genes, which are associated with amino acid transportation and biosynthesis, redox homeostasis, autophagy, cell survival, and cell death to maintain cellular homeostasis [8,9].

ATF4 is also the major regulator of mitochondrial stress through the ISR pathway [10]. Mitochondria are the key organelles that regulate energy metabolism and produce ATP through oxidative phosphorylation (OXPHOS) or glycolysis [11]. In mitochondria stress conditions, cancer cells upregulate some enzymes that are associated with glutathione (GSH) synthesis and amino acid transporters, including xCT (SLC7A11) [10]. The xCT is the light chain subunit of the cystine/glutamate antiporter at the plasma membrane. The function of xCT is exporting intracellular glutamate by exchanging extracellular cystine to maintain the synthesis of GSH. These reveal that the mitochondrial stress response is involved in redox balance.

The high expression of xCT protects cancer cells from oxidative stress and promotes resistance to cancer treatment through the reactive oxygen species(ROS)-activated ATF4–xCT pathway [12,13,14]. In conditions where glucose is limited, cancer cells utilize amino acids, especially glutamine, as the carbon and nitrogen sources for tricarboxylic acid (TCA) cycle and GSH production to maintain cellular biosynthesis and balance the levels of intracellular ROS [15,16]. However, during glucose deprivation, the upregulation of xCT attenuates nutrient flexibility and exacerbates cell death through the dysregulation of redox balance by exporting glutamate [17,18]. Further understanding the mechanism of the anticancer effect via xCT during glucose deprivation is important when developing anticancer therapies.

Salubrinal is the selective inhibitor of eIF2α dephosphorylation and can activate ISR through increasing the level of phosphorylated eIF2α (p-eIF2α) [19]. The role of ISR in tumor development is still controversial. ISR-related proteins were found to be more highly expressed in tumor tissue specimens from colon, lung, and breast cancer than in a specimen of normal tissue [20]. The activation of ISR is also associated with tumor growth [21], epithelial-to-mesenchymal transition, tumor invasion [22], and chemo-resistance [12] and is suspected of promoting tumor development. However, ISR-induced apoptosis has been reported to limit tumor development in lung cancer cells [23]. The dual role of ISR has also been observed in epidermoid carcinoma growth and in medulloblastoma tumorigenesis [24,25].

When developing anticancer therapy which targets glucose metabolism, the matter of how cancer cells respond to this metabolic stress condition needs to be evaluated. Further understanding the relationship between ISR, xCT, metabolic flexibility, and oxidative stress during glucose deprivation in cancer cells is essential. In this study, we investigated the response of cancer cells to salubrinal treatment during glucose deprivation. The relationship between salubrinal and redox balance was also evaluated.

## 2. Materials and Methods

### 2.1. Cell Culture

Several cancer cell lines were used in study, including human breast cancer (MDA-MB-231, MCF-7, and Hs578t), human gastric cancer (AGS and NUGC3), and oral squamous cell carcinoma (SAS, SAS-M5, and OECM1). SAS-M5 is a metastatic SAS cell line that was previously established from metastatic pulmonary tumors by injecting highly tumorigenic SAS cells into the tail vein of nude mice [26]. Breast cancer and oral cancer cell lines were grown in Dulbecco’s Modified Eagle’s Medium (DMEM; Gibco, Grand Island, NY, USA (12800017)). Gastric cancer cell lines were grown in RPMI 1640 medium (Gibco, Grand Island, NY, USA (31800022)). DMEM and RPMI medium were prepared according to the description of previous studies [18,27]. Either glucose-free DMEM (11966025) or glucose-free RPMI (11879020) (Gibco, Grand Island, NY, USA) with or without additional supplementation of glucose (25 mM) (Gibco, Grand Island, NY, USA (11966-025)) was used in the glucose deprivation experiments. In the salubrinal experiments, the cells were pre-treated with salubrinal (30 μM) (Sigma-Aldrich, St. Louis, MO, USA (SML0951-25 mg)) for 24 h.

### 2.2. Western Blot Analysis

Cells were lysed in radioimmunoprecipitation assay lysis (RIPA) buffer. The cell lysate was collected after centrifugation at 13,000× *g* for 15 min. Proteins (20 μg) from cell lysates were separated by 8–12% sodium dodecyl sulfate-polyacrylamide gel electrophoresis and transferred onto polyvinylidene difluoride membranes (BiotraceTM, PALL Life sciences, Ann Arbor, MI, USA (BSP0161)). Signals after immunoblotting with primary and secondary antibodies overnight on the sample membrane were detected by a chemiluminescence kit (Immobilon Western Chemiluminescence HRP Substrates, Merck-Millipore, Billerica, MA, USA (WP20005)). A luminescence/fluorescence imaging system (GE Healthcare) and multi-gauge image analysis software version 3.0 (Fujifilm, Stockholm, Sweden) were used to analyze the image. The preparation of the RIPA buffer and detailed procedure has been described in a previous study [27]. The antibodies against α-tubulin (62204), p-eIF2α (44728G), eIF2α (AHO0802) (InvitrogenTM, Thermo Fisher Scientific, Carlsbad, CA, USA), ATF4 (Proteintech, Rosemont, IL, USA (10835-1-AP)), and xCT (Cell Signaling Technology, Beverly, MA, USA (#12691)) were used in this study.

### 2.3. Sulforhodamine B (SRB) Assay for Cell Growth

The cell growth was analyzed using a Sulforhodamine B (SRB) assay. Cells were seeded in 96-well cell culture plates (Greiner bio-one, Frickenhausen, Germany (655180)) at a density of 3–5 × 10^3^ cells per well and cultured for 24 h prior to the drug treatment. At the planned time point, cells were fixed with 10% (*w*/*v*) trichloroacetic acid (TCA) at 4 °C for 1 h and stained with SRB for 30 min. After washing repeatedly with 1% (*v*/*v*) acetic acid (J.T baker, Center Valley, PA, USA (9508-03)) to remove excess dye, the protein-bound dye was dissolved in 10 mM of Tris base (J.T baker, Center Valley, PA, USA (4109-06)) solution for OD determination at 510 nm using a microplate reader. SRB (S1402) and TCA (T0699) were purchased from Sigma-Aldrich (St. Louis, MO, USA).

### 2.4. Clonogenic Assay

MDA-MB-231 (200 cells/well), AGS (500 cells/well) and SAS (100 cells/well) cells were cultured in a 6-well culture plate (Greiner bio-one, Frickenhausen, Germany (657160)). At a certain time point, cells were washed in 1× phosphate-buffered saline (PBS), fixed with 1 mL methanol (Merk, Darmstadt, Germany (1.07018.2511)) at 4 °C for 10 min, and then stained by 1 mL 0.01% crystal violet (Sigma-Aldrich, St. Louis, MO, USA (C3886)) for 30 min. The crystal violet was washed by ddH_2_O and dried in the room air. The colony was defined as a cellular group with more than 50 cells. The number of colonies present was counted under the microscope.

### 2.5. Transwell Migration Assay

Cells (1 × 10^5^ cells/well) were cultured in a transwell insert with a polycarbonate membrane (8 μm pore size) in a 24-well transwell plate (Falcon, Corning incorporated, One Riverfront Plaza, NY, USA (353504)). At a certain time point, the transwell insert was fixed by methanol at 4 °C for 20 min and then stained by Liu’s stain A (Tonyar biotech, Taoyuan, Taiwan (03R011)) for 5 min and Liu’s stain B (Tonyar biotech, Taoyuan, Taiwan (03R021)) for 30 min at room temperature. After washing with ddH_2_O to remove excess dye, the transwell inserts were dried in the room’s air. The number of migrated cells was counted using a photograph taken by the microscope.

### 2.6. Propidium Iodide (PI) Exclusion Assay

The cells (2 × 10^5^ cells/well) were seeded in a 6-well culture plate. The next day, the medium was changed, and we added different treatments according to the study design. After 24 h, the cells were collected and re-suspended in PBS with 5 mg/mL of PI (Sigma-Aldrich, St. Louis, MO, USA (P4170)). The PI fluorescence intensity of at least 20,000 cells at FL1 was determined by flow cytometry (FACS Calibur flow cytometer; Becton Dickinson, NJ, USA)). The data were evaluated by the Cell Quest software (Becton Dickinson). N-acetylcysteine (NAC, A0737), sulfasalazine (S0883), and dimethyl-α-ketoglutarate (dm-αKG, 349631) were purchased from Sigma-Aldrich (St. Louis, MO, USA).

### 2.7. Intracellular ROS and Mitochondrial ROS Measurement

The cells (2 × 10^5^ cells/well) were seeded in a 6-well culture plate. The next day, the medium was changed, and we added different treatments according to the study design. After 24 h, the cells were incubated with 10 μM of dichlorodihydro-fluorescein diacetate (DCFH-dA) for 30 min or 10 μM of MitoSOX Red for 10 min. Then, the cells were collected and re-suspended in PBS. The DCF fluorescence intensity of at least 20,000 cells at FL1 and the MitoSOX Red fluorescence intensity at FL2 were determined by flow cytometry. The data were evaluated by the Cell Quest software. The DCF (D399) and MitoSOX Red (M36008) were purchased from Molecular ProbesTM, InvitrogenTM, and Thermo Fisher Scientific (Eugene, OR, USA).

### 2.8. Small Interfering RNA (siRNA)-Mediated Genetic Knockdown

The cells (2 × 10^5^ cells) were seeded in a 6-well culture plate. The next day, the culture medium was changed to antibiotic-free medium. The siRNA-lipid complex was prepared using a mixture of lipofectamine RNAi MAX reagent (Invitrogen^TM^, Thermo Fisher Scientific, Carlsbad, CA, USA (13778100)) and the indicated concentration of siRNA in antibiotic/serum-free medium for 5 min at room temperature. The cells cultured in the antibiotic-free medium were treated with a siRNA-lipid complex for 48 h and then collected for further experiments. We performed Western blotting to confirm the effect of siRNA-mediated genetic knockdowns. Non-target (scramble (D-001810-01-05)) and xCT siRNAs (L-007612-01) were purchased from GE Healthcare Dharmacon (Lafayette, CO, USA).

### 2.9. Intracellular Glutamate Measurement

The cells were seeded in a 10 cm dish at a density of 6 × 10^5^ cells. The next day, the medium was changed according to the study design with different treatments. After 1 h, the intracellular level of glutamate was determined using a Glutamate Colorimetric Assay Kit (BioVision, Milpitas, CA, USA (K629)) according to the manufacturer’s protocol.

### 2.10. Statistical Analysis

The data are presented as the means ±SEMs of the results from three independent experiments. The statistical significance of the differences between the two groups was analyzed by Student’s *t*-test (GraphPad PRISM software version 6, GraphPad Software, La Jolla, CA, USA). The significance level was set at less than 0.05.

## 3. Results

### 3.1. Salubrinal Activated ISR and Inhibited Cell Growth and Migration

**Figure 1:**
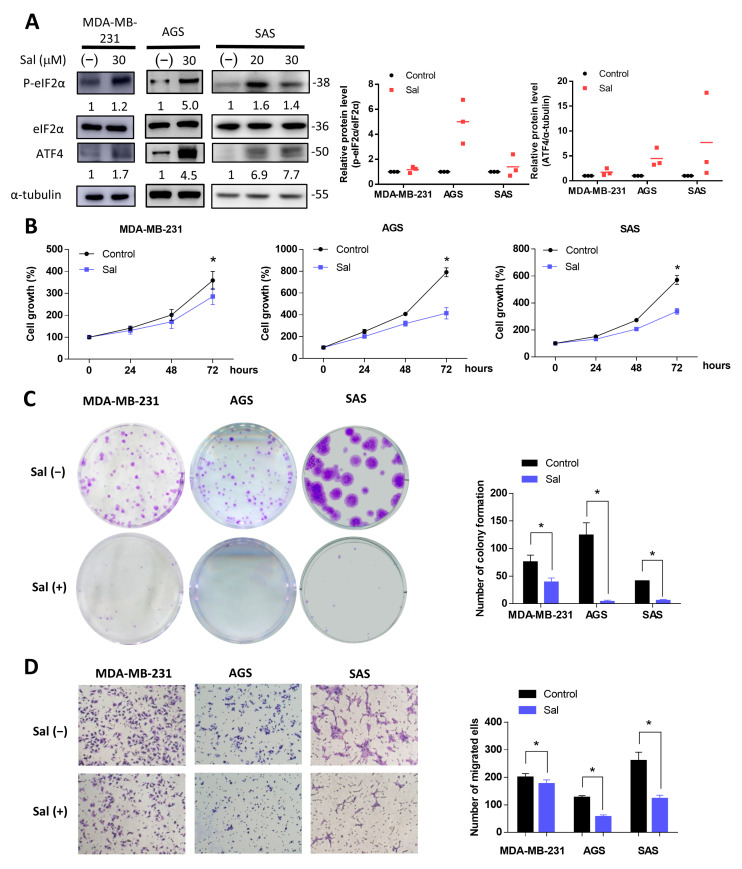
Salubrinal inhibited cell growth and migration in cancer cells. Three cancer cell lines (MDA-MB-231, AGS, and SAS) were treated with or without 30 μM of salubrinal in normal culture medium. (**A**) The protein levels of eIF2α, p-eIF2α, and ATF4 were detected using Western blotting after salubrinal treatment for 24 h; The value of the control group was set as 1. (**B**) The cell growth rate at 0, 24, 48, and 72 h after salubrinal treatment was detected by a sulforhodamine B (SRB) assay. (**C**) The colony formation ability was checked after treatment with or without salubrinal for 10 days in MDA-MB-231 cells, for 7 days in AGS cells, and for 9 days in SAS cells. (**D**) The ability of cell migration was detected using a transwell migration assay. After salubrinal pre-treatment for 24 h, the migration time was found to be 6 h for MDA-MB-231 cells, 8 h for AGS cells, and 24 h for SAS cells. The photograph was taken by a microscope (100× magnification). The data are presented as the means ±SEMs of the results from three independent experiments. * *p* < 0.05. Sal: salubrinal.

We first detected the effect of salubrinal on the ISR-related proteins of three cancer cell lines, MDA-MB-231 (breast cancer), AGS (gastric cancer), and SAS (squamous cell carcinoma of the tongue). The activation of ISR is associated with increased levels of p-eIF2α and ATF4, the main effector of the ISR. The salubrinal increased the protein levels of p-eIF2α and ATF4 in these three cancer cell lines (Figure 1A). Therefore, we used salubrinal as the activator of ISR and evaluated the effect of salubrinal on cell growth, colony formation, and migration in glucose-rich conditions. The results showed that salubrinal decreased the cell growth, the number of colonies formed, and migration ability when cells were cultured in the glucose-containing medium (Figure 1B–D). Although statistically significant, MDA-MB-231 cells did not show as decreased migration as the AGS and SAS cell lines after salubrinal treatment (Figure 1D). The activation of ISR inhibits the translation of cap-dependent mRNA to attenuate protein synthesis but upregulates the expression of ATF4 and the downstream stress response genes to maintain cellular homeostasis [8]. ISR also induces cell death if cells do not adapt to the stress [8]. These inhibitory effects of salubrinal on cell growth, migration, and colony formation might be protein-synthesis-attenuation- or cell death-related. We then evaluated the effects of salubrinal on cell death.

### 3.2. Salubrinal Increased Cell Death Rate and ROS Levels in Cancer Cells under Glucose Deprivation

**Figure 2:**
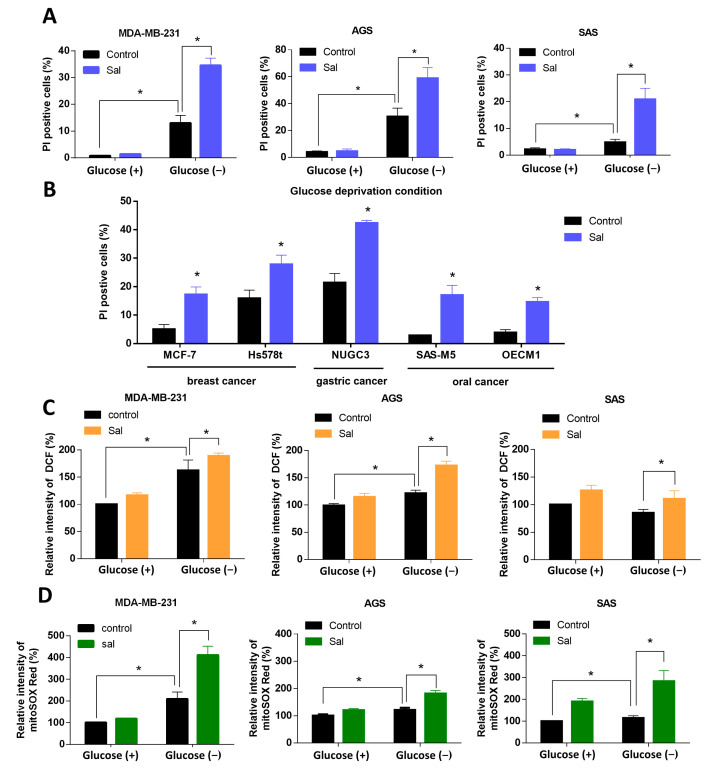
Salubrinal enhanced glucose deprivation-induced cell death and ROS levels. The cells were treated according to the study design with or without 30 μM of salubrinal for 24 h except for 48 h in OECM1. (**A**,**B**) The cell death rate was evaluated by a propidium iodide (PI) exclusion assay in MDAMB-231, AGS, and SAS cells (**A**), as well as in other cell lines during glucose deprivation (**B**). (**C**,**D**) The levels of intracellular ROS (icROS) and mitochondrial ROS (mtROS) were detected using flow cytometry with DCFH-dA staining (**C**) and mitoSOX Red dye (**D**). The measured value of ROS from the control group under glucose-containing conditions was normalized to 100%. The data are presented as the means ±SEMs of the results from three independent experiments. * *p* < 0.05. Sal: salubrinal. Glucose (+): medium with 25 mM of glucose. Glucose (−): glucose-free medium.

The cell death rate was evaluated by PI stain through flow cytometry. The salubrinal did not obviously change the cell death rate in glucose-rich conditions. However, co-treatment with salubrinal and glucose deprivation induced a significantly higher cell death rate of 20–60% than treatment with glucose deprivation only (Figure 2A). This synergic cytotoxic effect was also observed in the other cell lines of breast, gastric, and oral cancer, including MCF-7, Hs578t, NUGC3, SAS-M5, and OECM1 (Figure 2B). The results revealed that the activation of ISR by salubrinal increases sensitivity to glucose deprivation in breast, gastric, and oral cancer cells lines. Then, we chose the MDA-MB-231, AGS, and SAS cell lines as the representative cell lines for breast, gastric, and oral cancer to evaluate the mechanism underlying the salubrinal-enhanced cell death under glucose deprivation.

According to our previous study [18], glucose-deprivation-induced-cell death was associated with a dysregulation of ROS. We then evaluated if salubrinal also affected the redox balance during glucose deprivation. After a co-treatment involving salubrinal and glucose deprivation, the levels of intracellular ROS (icROS) increased in MDA-MB-231 and AGS cell lines but not SAS cell lines (Figure 2C). The levels of mitochondrial ROS (mtROS) increased in all three cancer cell lines (Figure 2D). Salubrinal significantly increased the levels of mtROS both in glucose-containing conditions and in glucose-deprivation conditions (Figure 2C,D). The ROS levels were found to be highest in the groups co-treated with salubrinal and glucose deprivation. The results showed that salubrinal enhances glucose-deprivation-induced cell death and glucose-deprivation-increased mtROS levels.

### 3.3. ROS Were Involved in Salubrinal-Enhanced Cell Death during Glucose Deprivation

**Figure 3:**
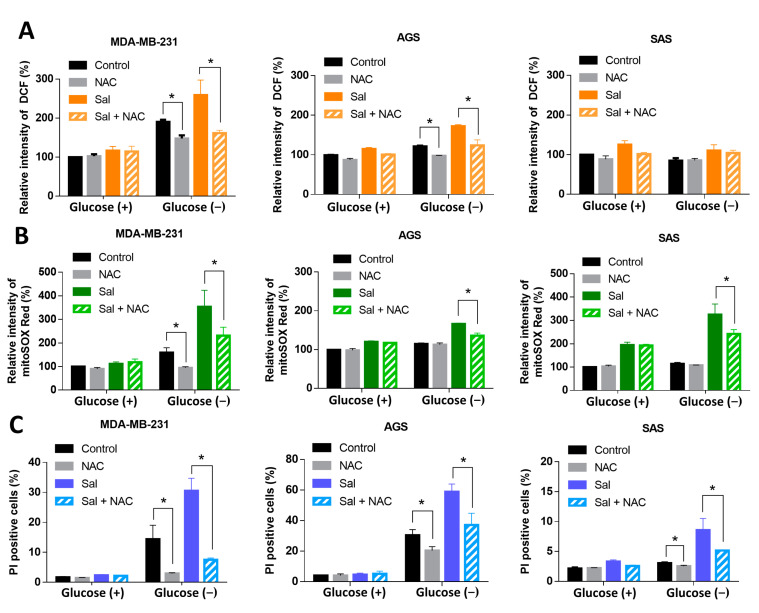
ROS involved in salubrinal enhanced-cell death rate during glucose deprivation. The cells were treated according to the study design with or without 30 μM of salubrinal and/or 1 mM of N-acetyl-cysteine (NAC) for 24 h. (**A**,**B**) The levels of intracellular ROS (icROS) and mitochondrial ROS (mtROS) were detected using flow cytometry with DCFH-dA staining (**A**) and mitoSOX Red dye (**B**). The measured value of ROS from the control group under glucose-containing conditions was normalized to 100%. (**C**) The cell death rate was evaluated by a PI exclusion assay. The data are presented as the means ±SEMs of the results from three independent experiments. * *p* < 0.05. NAC: N-acetyl-cysteine. Sal: salubrinal. Glucose (+): medium with 25 mM of glucose. Glucose (−): glucose-free medium.

To determine if ROS were involved in the salubrinal-enhanced cell death during glucose deprivation, we used a ROS scavenger, N-acetylcysteine (NAC), to reduce oxidative stress and check the response of the cells. NAC was found to reduce the levels of icROS in MDA-MB-231 and AGS cell lines but not those of the SAS cell line during glucose deprivation (Figure 3A). NAC reduced the levels of mtROS, which are enhanced by co-treatment with salubrinal and glucose deprivation (Figure 3B). NAC also attenuated the glucose deprivation-induced cell death in all MDA-MB-231, AGS, and SAS cell lines with or without co-treatment with salubrinal (Figure 3C). In glucose-containing conditions, NAC did not affect the cell death rate or the levels of icROS and mtROS in cells with salubrinal treatment (Figure 3A–C). The results suggested that mitochondrial oxidative stress is involved in salubrinal-enhanced cell death during glucose deprivation.

### 3.4. Salubrinal Promoted Cell Death during Glucose Deprivation through the Upregulation of xCT

xCT is the light chain subunit of cystine/glutamate antiporter and regulates oxidative stress by maintaining GSH synthesis [28]. The upregulation of xCT was also reported to reduce the nutrient flexibility in cancer cells [17,18]. The role of xCT in salubrinal-enhanced cell death during nutrient deficiency is still poorly understood.

**Figure 4:**
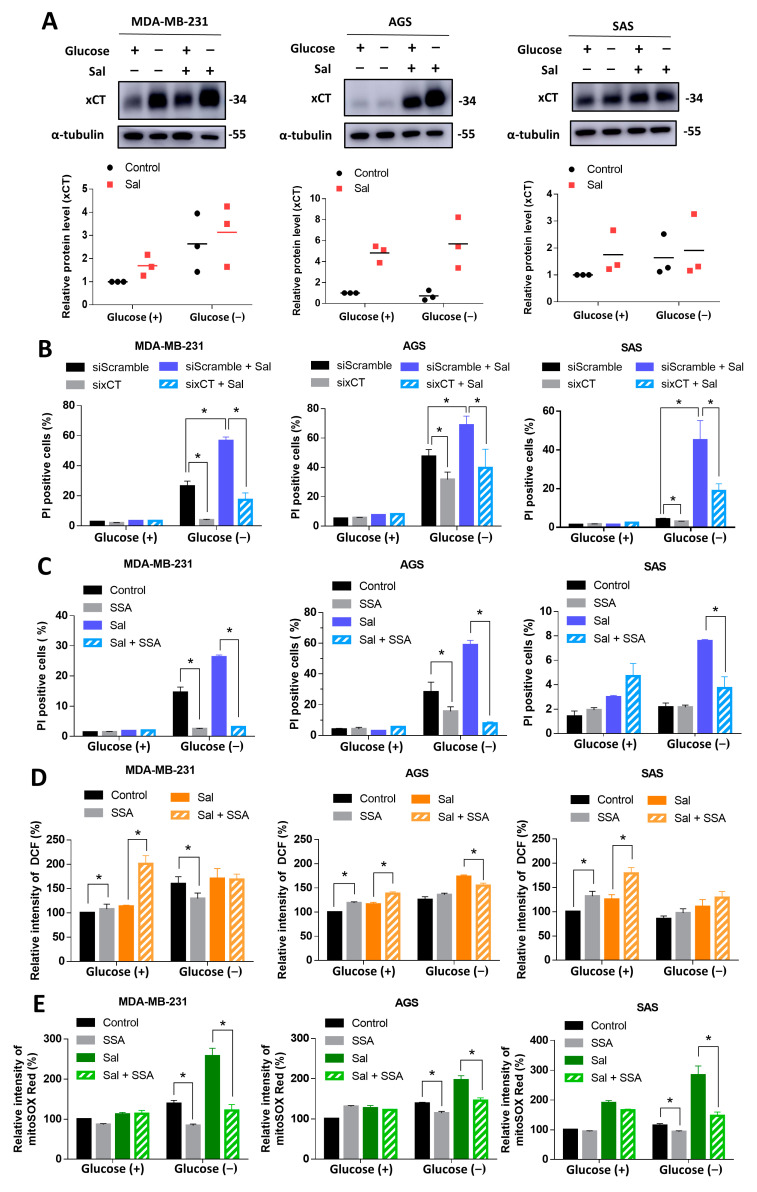
Salubrinal enhanced cell death during glucose deprivation through the upregulation of xCT. The cells were treated according to the study design with or without 30 μM of salubrinal, siRNAs against xCT (sixCT), or 350 μM of sulfasalazine for 24 h. (**A**) The protein levels of xCT were detected using Western blotting. The value of the control group under glucose-containing conditions was set as 1. The cell death rate was evaluated by a PI exclusion assay after the knockdown of xCT by sixCT (**B**) or the supplementation of sulfasalazine (**C**). The levels of intracellular ROS (icROS) and mitochondrial ROS (mtROS) were detected using flow cytometry with DCFH-dA staining (**D**) and mitoSOX Red dye (**E**). The value of ROS measured from the control group under glucose-containing conditions was normalized to 100%. The data are presented as the means ±SEMs of the results from three independent experiments. * *p* < 0.05. SSA: sulfasalazine. Sal: salubrinal. Glucose (+): medium with 25 mM of glucose. Glucose (−): glucose-free medium.

To determine whether xCT is involved in cell death and mtROS levels, which were both increased by salubrinal during glucose deprivation, we evaluated the protein levels of xCT in MDA-MB-231, AGS, and SAS cells with or without salubrinal treatment. The results revealed that salubrinal enhanced the protein levels of xCT (Figure 4A). The knockdown of xCT by siRNA and supplementation with the xCT inhibitor, sulfasalazine [29], significantly attenuated the glucose deprivation-induced cell death in cancer cells with or without salubrinal treatment in MDA-MB-231, AGS, and SAS cell lines (Figure 4B,C). In glucose-containing conditions, the inhibition of the function of xCT by sulfasalazine increased the levels of icROS but did not increase the cell death rate (Figure 4C,D). This revealed that the cells could tolerate higher levels of ROS under nutrient-rich conditions. When co-treating with salubrinal and glucose deprivation, the sulfasalazine did not obviously affect the levels of icROS but significantly decreased the levels of mtROS in cancer cells (Figure 4D,E). These findings provided evidence that xCT is involved in salubrinal-enhanced cell death during glucose deprivation through the imbalance of mtROS.

### 3.5. Dm-αKG Rescued the Salubrinal-Enhanced Cell Death during Glucose Deprivation

**Figure 5:**
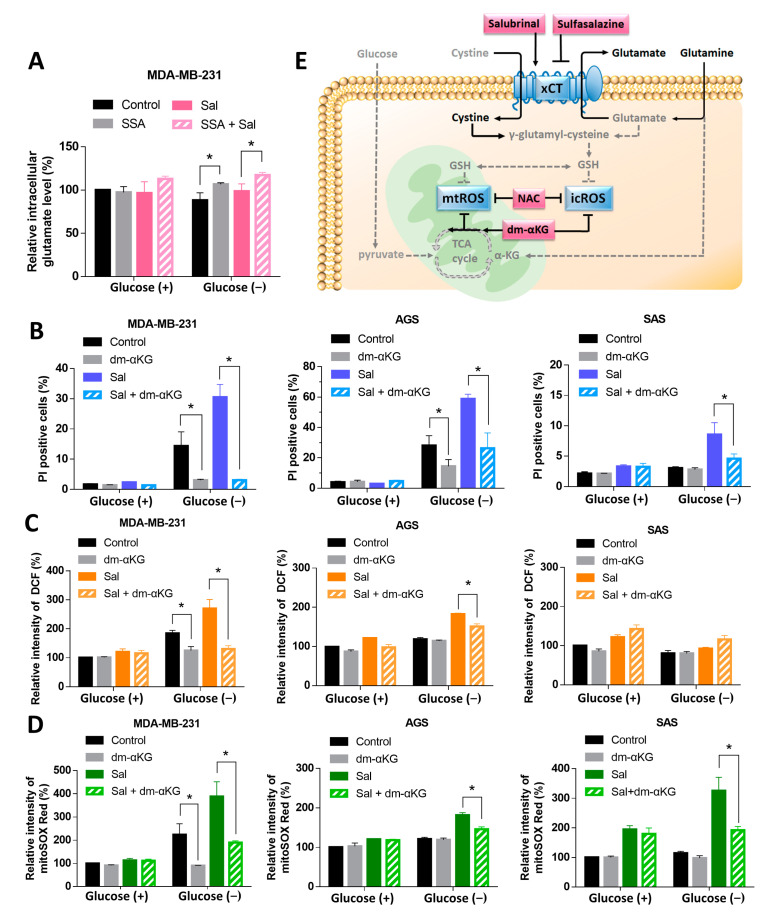
Dm-αKG improved the salubrinal-enhanced cell death and decreased the levels of mtROS during glucose deprivation. (**A**) Intracellular glutamate levels of MDA-MB-231 were detected using a glutamate assay kit. The measured level of glutamate in the control group under glucose-containing conditions was normalized at 100%. The cells were treated according to the study design with or without 30 μM of salubrinal and/or 4 mM of dm-αKG for 24 h. (**B**) The cell death rate was evaluated by a PI exclusion assay. The levels of intracellular ROS (icROS) and mitochondrial ROS (mtROS) were detected using flow cytometry with DCFH-dA staining (**C**) and mitoSOX Red dye (**D**). The measured value of ROS from the control group under glucose-containing conditions was normalized as 100%. (**E**) Model of the mechanism of salubrinal enhanced-cell death during glucose deprivation. Salubrinal and glucose deprivation synergic increased the levels of mtROS through enhancing the expression of xCT with nutrient deficiency-related oxidative stress. Sulfasalazine, NAC, and dm-αKG were found to reverse salubrinal enhanced-cell death during glucose deprivation. Dash line arrows means the attenuation effects when glucose deprivation. Solid line arrows indicates the enhanced effects when glucose deprivation. T shape lines means inhibitory effects when treating with sulfasalazine, NAC, and dm-αKG. The data are presented as the means ±SEMs of the results from three independent experiments. * *p* < 0.05. dm-αKG: dimethyl-α-ketoglutarate. Sal: salubrinal. Glucose (+): medium with 25 mM of glucose. Glucose (−): glucose-free medium. GSH: glutathione. icROS: intracellular ROS. mtROS: mitochondrial ROS. NAC: N-acetyl-cysteine.

We then investigated how salubrinal promoted cell death and ROS levels through the upregulation of xCT during glucose deprivation. Cancer cells used glutamine as a substitute to maintain the cellular biosynthesis and redox balance when glucose is limited [16]. Glutamine can be converted into glutamate and α-ketoglutarate (α-KG) to fuel the TCA cycle. The upregulation of xCT decreased the levels of intracellular glutamate in order to exchange the extracellular cystine. We hypothesized that the glutamate deficiency was involved in the salubrinal-increased cell death and ROS levels. The intracellular glutamate measurement revealed that the glutamate level was not significantly decreased by salubrinal. However, sulfasalazine with or without salubrinal treatment was found to increase the intracellular glutamate level under glucose deprivation (Figure 5A). Glutamate is a source of α-KG and could be utilized to maintain the TCA cycle. The supplementation of dm-αKG significantly reduced the salubrinal-enhanced cell death and salubrinal-increased mtROS levels during glucose deprivation (Figure 5B,D). The levels of icROS were decreased when co-treating with salubrinal and dm-αKG under glucose deprivation in the MDA-MB-231 and AGS cell lines, but not in the SAS cell line (Figure 5C). These results support the notion that salubrinal-enhanced cell death and salubrinal-increased mtROS levels during glucose deprivation are associated with glutamate deficiency. This demonstrates the role of mtROS in cell survival under nutrient-limited conditions.

## 4. Discussion

This study demonstrated that salubrinal enhanced glucose deprivation-induced cell death in breast, gastric, and tongue cancer cell lines through the upregulation of xCT and elevated ROS levels, especially mtROS (Figure 5E). The supplementation of dm-αKG or NAC as well as the inhibition of the function of xCT by siRNA or sulfasalazine could rescue cells from salubrinal enhanced-cell death during glucose deprivation (Figure 5E).

Salubrinal is a selective inhibitor of eIF2α dephosphorylation and activates ISR by increasing the levels of p-eIF2α. The initial effect of ISR is a reduction in global protein synthesis to release ER stress due to the decreased accumulation of unfolded protein [19,30]. In glucose-containing conditions, salubrinal inhibits cell growth, migration, and clonogenic formation but does not increase the cell death rate or ROS levels in breast, gastric, or tongue cancer cell lines (Figure 1B–D and Figure 2A,C,D). These results revealed that the ISR activated by salubrinal in the condition of sufficient nutrients only diminished cellular function due to the reduction in global protein synthesis but did not lead to oxidative stress and cell death. The activated ISR could keep cells in a cell cycle arrest status through the attenuation of cyclin D1 and restore cellular homeostasis to prevent stress-induced apoptosis [31,32].

Cancer cells use glucose as a major nutrient source and are highly dependent on glycolysis [1,33,34]. A lack of glucose induces autophagy or cell death through oxidative stress and ER stress [18,35,36,37]. Cancer cells have a strong detoxification activity against ROS to adapt to intracellular oxidative stress and the hypoxic tumor microenvironment. Superoxide dismutases, catalase, the thioredoxin system, and the GSH system are all involved in the redox balance in cells [38]. Glucose deprivation decreases the synthesis of NADPH by limiting the substrates of the pentose phosphate pathway in glycolysis. An insufficient level of NADPH can not provide sufficient reduction power to support the GSH cycle. This might contribute to the fact that glucose deprivation increases oxidative stress in cancer cells. Salubrinal further enhances the cell death rate and the levels of mtROS during glucose deprivation (Figure 2A–D). The ROS scavenger, NAC, was found to reduce the mitochondrial oxidative stress and rescued cells from salubrinal-enhanced cell death in all three cell lines (Figure 3B,C). These results demonstrated that mtROS were involved in salubrinal-enhanced cell death during glucose deprivation.

Salubrinal, as an activator of ISR, not only reduces global protein synthesis but also upregulates ATF4. ATF4 is a transcription factor that is associated with amino acid metabolism and redox regulation. ATF4 regulates several genes that are involved in GSH synthesis, including glycine transporter, xCT, glutamate-cysteine ligase catalytic and modifier subunit, thioredoxin reductase, and methylenetetrahydrofolate dehydrogenase [7,39]. In the mitochondrial stress condition, the upregulation of the enzymes related to the synthesis of amino acids, GSH, and amino acids transporters, including xCT, was also observed through the ISR-ATF4 pathway [10]. This suggested that one of the responses of cells to ISR is to increase the production of GSH to reduce oxidative stress. Our results also revealed that salubrinal does not affect ROS levels in conditions with sufficient nutrients. Inhibiting the function of xCT was found to increase the levels of icROS but not to drastically increase the cell death rate in the glucose-containing medium (Figure 4B–D). These results revealed that the redox balance system could still work normally in the condition of sufficient nutrients even with the limited synthesis of GSH.

On the other hand, during glucose deprivation, salubrinal increases the levels of ROS and leads to cell death. The knockdown of xCT or supplementation of sulfasalazine significantly decreases salubrinal-enhanced cell death and salubrinal-increased mtROS levels (Figure 4A–E). These results demonstrate that salubrinal with glucose deprivation leads to redox balance collapse, especially in the mitochondria, and therefore induces cell death. Increased levels of mtROS suggest that mitochondria are dysfunctional and under stress conditions. The cells under mitochondrial stress responses upregulate the genes and proteins related to GSH synthesis but have a decreased GSH/oxidized GSH (GSSG) ratio [10]. Increased consumption of GSH due to oxidative stress was suspected [10].

Not only the deficiency of ROS scavengers but also nutrient deficiency were found to increase mtROS levels. In low-glucose conditions, cancer cells upregulated OXPHOS to maintain metabolic homeostasis [40]. Cancer cells utilize amino acids as a substitute for fueling the TCA cycle [15], the synthesis of glycolytic intermediates [41], and maintaining the function of OXPHOS. A deficiency of amino acids during glucose deprivation would impair mitochondrial function due to the collapse of the TCA cycle and OXPHOS. The collapse of the TCA cycle leads to an increased oxidized/reduced nicotinamide adenine dinucleotide (NAD+/NADH) ratio and oxidized/reduced flavin adenine nucleotide (FAD+/FADH2) ratio. Decreased levels of NADH and FADH2 would limit the proton transport across the inner membrane and impair the proton gradient between the intermembrane space and the matrix of the mitochondria. In this study, the supplementation of dm-αKG could fuel the TCA cycle and decrease the salubrinal-enhanced cell death and the salubrinal-increased mtROS levels during glucose deprivation (Figure 5B–D). These results support the notion that cancer cells depend more on amino acids to maintain mitochondrial function under glucose-deprivation conditions. Any factors such as salubrinal or the upregulation of xCT that lead to greater consumption of amino acids would contribute to mitochondrial dysfunction and cell death.

Cancer cells prefer aerobic glycolysis as the major metabolism pathway. Targeting glycolytic metabolism as a monotherapy has shown mixed favorable and disappointing results for suppressing cancer progression in pre-clinical studies and has not been used in clinical practice [42]. Concerns regarding the use of glycolytic inhibitors—for example, 2-deoxy-D-glucose—in cancer treatment as a monotherapy include their hypoglycemia-like symptoms and the impairment of function in the heart and brain [43,44]. Due to metabolic flexibility in cancer, combined therapy of glycolytic inhibitor and other agents has shown synergic antitumor effects and lower toxicity [45,46,47,48]. Our results also showed the consistent finding that glucose deprivation with salubrinal induces cell death in breast, gastric, and tongue cancer cell lines more than glucose deprivation treatment alone.

The role of salubrinal in cancer treatment is still controversial. Salubrinal activates ISR through inhibiting eIF2α dephosphorylation and protects cells from ER stress-mediated apoptosis [19]. Salubrinal also enhances cisplatin resistance in gastric cancer through xCT upregulation [27]. Salubrinal alone shows cytotoxicity in inflammatory breast cancer cells [49] but not in melanoma [50], breast (MDA-MB-231), gastric (AGS), or tongue (SAS) cancer cell lines (Figure 2A). On the other hand, salubrinal has a synergic antitumor effect when combined with a cap-dependent translation inhibitor, proteasome inhibitor, or doxorubicin [50,51,52,53]. When targeting glucose metabolism, rapamycin, which inhibits glycolysis through suppressing the mTOR pathway, also shows synergistic antitumor activity with salubrinal in cholangiocarcinoma cells [54].

The other activators of ISR also show cytoprotection or cell destruction under different conditions. L-asparaginase, arginine deiminase, and halofuginone all activate ISR through amino acid starvation response and show anticancer activity [8,55,56,57,58]. Under nutrient-rich conditions, halofuginone induced autophagic flux and inhibited glycolysis in colorectal cancer cells. However, under nutrient-poor conditions, halofuginone inhibited autophagy and gluconeogenesis [58]. The eIF2α dephosphorylation inhibitor, nelfinavir, showed synergic antitumor effects when combined with glycolytic inhibitor, ritonavir, and metformin through ER stress and mtROS-mediated autophagy [59,60,61].

The ISR is a complex adaptive response that helps cells to survive different stresses but also induces cell death when cells can not restore homeostasis [8]. This may explain why both pro-survival and pro-death roles of ISR were observed in cancer cells. This also limited the application of the activators and inhibitors of ISR in cancer treatment. In this study, salubrinal showed a synergic antitumor effect when combined with glucose deprivation through ROS-mediated cell death. Several studies have also demonstrated similar antitumor effects when combining ISR activators with glycolysis inhibitors, including rapamycin, ritonavir, and metformin [59,60,61]. Targeting glucose metabolism by a direct glycolysis pathway inhibitor or indirect inhibitor through other pathways, such as the mTOR or AMPK pathways, with ISR activators may be a useful direction for the further development of cancer therapy. Combined therapy also reduces the safety concerns about limited glucose metabolism in normal cells. Our study reveals a synergic anticancer effect through co-treatment with salubrinal and glucose deprivation. Combining therapy with salubrinal and glycolysis inhibitors to treat breast, gastric, and tongue cancers may be effective and is an important direction for future studies to investigate.

In conclusion, our results demonstrate that salubrinal effectively enhances the cell death rate during glucose deprivation through the upregulation of xCT and mitochondrial oxidative stress in breast, gastric, and tongue cancer. These findings improve our understanding of the interaction between salubrinal, the metabolism of amino acids, and mitochondrial oxidative stress during glucose deprivation in cancer cells. The regulation of mtROS is an important means for cancer cells to adapt to nutrient-limited conditions. Combining salubrinal with agents that impair glucose metabolism is suggested to have beneficial effects when targeting cancer cells with the upregulation of xCT.
